# Identification and Validation of Two Lung Adenocarcinoma-Development Characteristic Gene Sets for Diagnosing Lung Adenocarcinoma and Predicting Prognosis

**DOI:** 10.3389/fgene.2020.565206

**Published:** 2020-12-21

**Authors:** Cheng Liu, Xiang Li, Hua Shao, Dan Li

**Affiliations:** ^1^Department of Thoracic Surgery, The Fourth Affiliated Hospital of Harbin Medical University, Harbin, China; ^2^Department of Neurology, The Fourth Affiliated Hospital of Harbin Medical University, Harbin, China

**Keywords:** lung adenocarcinoma, prognostic stratification system, The Cancer Genome Atlas, gene set variation analysis score, predicting prognosis

## Abstract

**Background**: Lung adenocarcinoma (LUAD) is one of the main types of lung cancer. Because of its low early diagnosis rate, poor late prognosis, and high mortality, it is of great significance to find biomarkers for diagnosis and prognosis.

**Methods**: Five hundred and twelve LUADs from The Cancer Genome Atlas were used for differential expression analysis and short time-series expression miner (STEM) analysis to identify the LUAD-development characteristic genes. Survival analysis was used to identify the LUAD-unfavorable genes and LUAD-favorable genes. Gene set variation analysis (GSVA) was used to score individual samples against the two gene sets. Receiver operating characteristic (ROC) curve analysis and univariate and multivariate Cox regression analysis were used to explore the diagnostic and prognostic ability of the two GSVA score systems. Two independent data sets from Gene Expression Omnibus (GEO) were used for verifying the results. Functional enrichment analysis was used to explore the potential biological functions of LUAD-unfavorable genes.

**Results**: With the development of LUAD, 185 differentially expressed genes (DEGs) were gradually upregulated, of which 84 genes were associated with LUAD survival and named as LUAD-unfavorable gene set. While 237 DEGs were gradually downregulated, of which 39 genes were associated with LUAD survival and named as LUAD-favorable gene set. ROC curve analysis and univariate/multivariate Cox proportional hazards analyses indicated both of LUAD-unfavorable GSVA score and LUAD-favorable GSVA score were a biomarker of LUAD. Moreover, both of these two GSVA score systems were an independent factor for LUAD prognosis. The LUAD-unfavorable genes were significantly involved in p53 signaling pathway, Oocyte meiosis, and Cell cycle.

**Conclusion**: We identified and validated two LUAD-development characteristic gene sets that not only have diagnostic value but also prognostic value. It may provide new insight for further research on LUAD.

## Introduction

Lung cancer is the most common cancer (11.6% of the total cases) among men and women in the world, which is also the main cause of cancer death (18.4% of the total cancer deaths; Bray et al., 2018). Non-small cell lung cancer (NSCLC) accounts for 85% of all lung cancer cases (Govindan et al., 2006), and lung adenocarcinoma (LUAD) is one of the main subtypes of NSCLC. Smoking is currently considered to be the main cause of lung cancer. However, LUAD is more likely to occur in women who do not smoke, and the age of patients tends to become younger (Hecht, 1999; Donner et al., 2018). Early, LUAD can be treated by surgery; however, most patients with LUAD are often diagnosed with advanced cancer (Ding et al., 2008). Although target therapy is effective for selected advanced LUAD, the overall survival of patients is poor due to the emergence of drug resistance. Therefore, it has become one of the hot spots in clinical research to find the diagnosis and prognosis indexes of LUAD.

In recent years, high-throughput sequencing technology and gene database have been widely used in the study of cancer diagnosis and prognosis (Feng et al., 2016; Dama et al., 2017; Zhao et al., 2018a; He et al., 2019). For example, EGFR, KRAS, BRAF, and ERBB 2 have been shown to be associated with treatment efficacy and prognosis (Naoki et al., 2002; Mendelsohn and Baselga, 2003; Guan et al., 2013). Moreover, DGCR 5 has been found to be a prognostic indicator and therapeutic target for the diagnosis and treatment of LUAD (Dong et al., 2018). Overexpression of Rcc 2 induces epithelial-mesenchymal metastasis in LUAD, enhances cell mobility, and promotes tumor metastasis (Pang et al., 2017). Overexpression of KIF20A confers malignant phenotype of LUAD by promoting cell proliferation and inhibiting apoptosis (Zhao et al., 2018b). However, most studies do not take the simultaneous changes of multiple genes into account. Moreover, there are few studies on the LUAD-development characteristic gene sets.

In present study, we identified two LUAD-development characteristic gene sets named as LUAD-unfavorable gene set and LUAD-favorable gene set. Gene set variation analysis (GSVA) was used to score individual samples against the two gene sets. Survival analysis and receiver operating characteristic (ROC) curve analysis were used to identify the diagnostic and prognostic capabilities of two gene sets GSVA score, respectively. Both of LUAD-unfavorable GSVA score and LUAD-favorable GSVA score were reliable biomarkers for diagnosing LUAD and independent biomarkers for predicting prognosis.

## Materials and Methods

**Figure 1:**
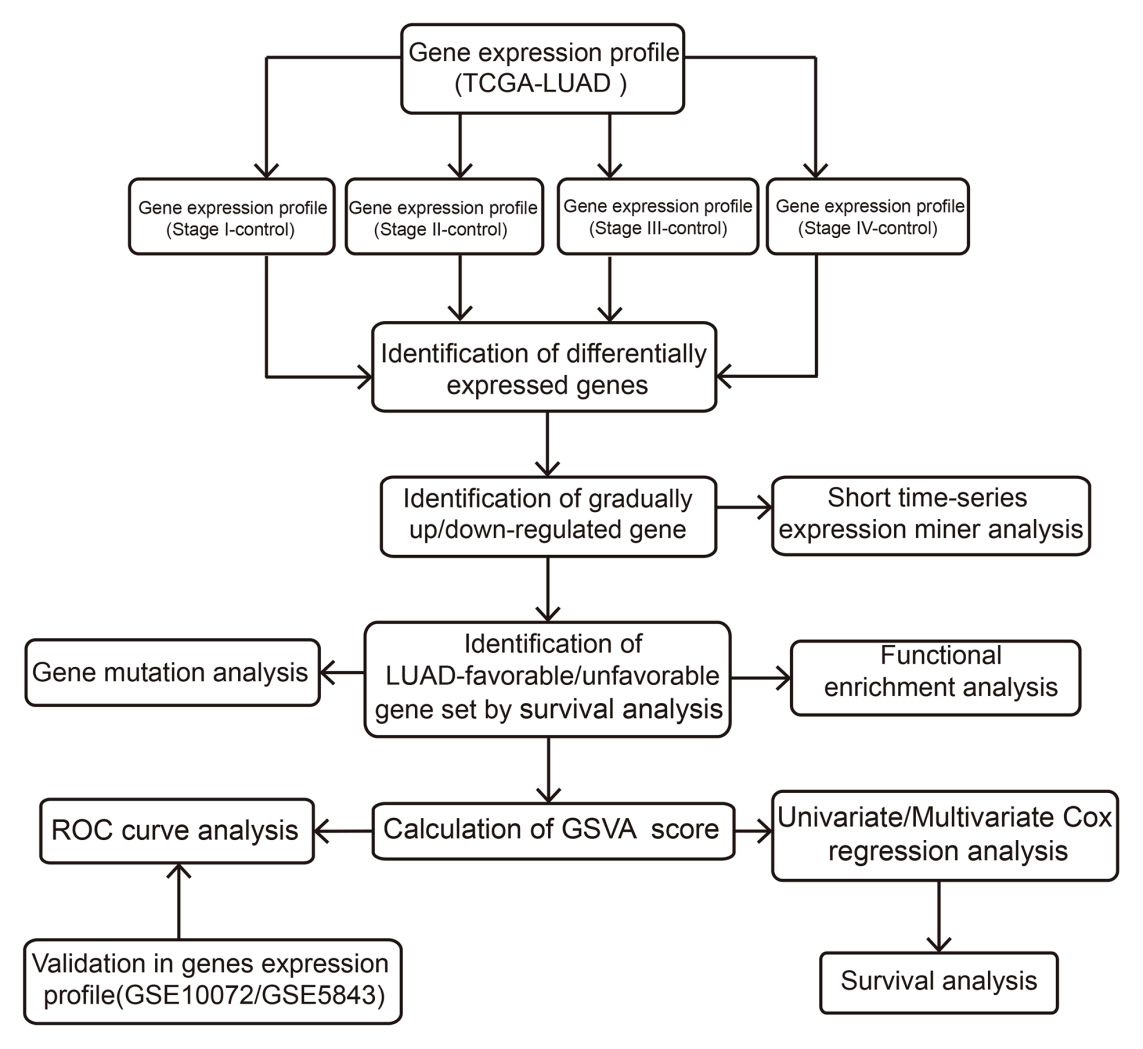
Flowchart of this study.

The Cancer Genome Atlas (TCGA; Tomczak et al., 2015)^1^ and Gene Expression Omnibus (GEO; Barrett et al., 2013)^2^ are the international genetic databases, which are publicly accessible and freely available to researchers. In our study, a total of 512 LUAD samples and 57 healthy lung tissue samples were downloaded from TCGA, including 281 stage I LUADs, 121 stage II LUADs, 84 stage III LUADs, and 26 stage IV LUADs. In addition, GSE10072 based on GPL96 platform was downloaded from GEO, including 58 LUAD samples and 49 healthy lung tissue samples. GSE31210 based on GPL570 platform was downloaded from GEO, including 226 LUAD samples and 20 healthy lung tissue samples. The two data sets were used to verify the prognostic value. The “normalizeBetweenArrays” function in the limma package (Ritchie et al., 2015) was used to normalize the gene expression profiles. If a gene responds to multiple probes, the average value of these probes is considered to be the expression value of the corresponding gene. The flow of this study is shown in Figure 1.

### Differential Expression Analysis and Short Time-Series Expression Miner

In TCGA, the RNA sequencing expression profile of LUAD was displayed as read counts, which was subsequently normalized by voom function (Law et al., 2014) in limma package. Differentially expressed genes (DEGs) in four stages of LUAD were identified using limma package, respectively. *p* < 0.01 adjusted by the false discovery rate (FDR) and |log fold change(FC)| > 1.5 were considered as significance. In the developing of LUAD, if a DEG was gradually upregulated (logFCstage I vs. control < logFCstage II vs. control < logFCstage III vs. control < logFCstage IV vs. control) or gradually downregulated (logFCstage I vs. control > logFCstage II vs. control > logFCstage III vs. control > logFCstage IV vs. control), and then it was considered to be LUAD-development characteristic gene. These genes were organized into different clusters based on expression patterns using short time-series expression miner (STEM; Ernst and Bar-Joseph, 2006).

### Survival Analysis and LUAD-Development Characteristic Gene Set

We used the median expression value of each LUAD-development characteristic gene as the cutoff point to dichotomize patients into high-expression group and low-expression group. Moreover, Kaplan Meier survival analysis and log rank method were performed to explore whether the expression level of the LUAD-development characteristic gene is related to the overall survival (OS) time. Survival analysis was performed using survival package^3^ in R, and *p* < 0.01 was considered as significance. In our study, a LUAD-development characteristic gene which gradually upregulated with the development of LUAD and associated with poor prognosis of LUAD was considered to be LUAD-unfavorable gene. On the contrary, a LUAD-development characteristic gene which gradually downregulated with the development of LUAD and associated with good prognosis of LUAD was considered to be LUAD-favorable gene. LUAD-unfavorable genes and LUAD-favorable genes constituted LUAD-unfavorable genes set and LUAD-favorable genes set, respectively.

### Calculation of LUAD-Development Characteristic GSVA Score

Gene set variation analysis is a popular method of scoring individual samples for molecular characteristics or gene sets. GSVA package (Hanzelmann et al., 2013) in R was used to calculate LUAD-unfavorable GSVA score and LUAD-favorable GSVA score for individual samples.

### ROC Curve Analysis and Univariate/Multivariate Cox Proportional Hazards Analyses

The pROC package (Robin et al., 2011) was used to conduct ROC curve analysis of LUAD-unfavorable GSVA score and LUAD-unfavorable GSVA score to evaluate their ability to diagnose LUAD. Univariate/multivariate Cox proportional hazards analyses were used to compare the relative prognostic value of the two GSVA score systems with that of routine clinicopathological features.

### Functional Enrichment Analysis

To further explore the biological function of LUAD-unfavorable genes, Gene Ontology (GO) and Kyoto Encyclopedia of Genes and Genomes (KEGG) pathway enrichment analysis were performed using the clusterProfiler package (Yu et al., 2012) in R. *p* < 0.05 was considered as significance.

### Gene Mutation Analysis and Validation of Differential Expression of LUAD-Unfavorable Genes at Protein Level

In order to explore the potential mechanism about differential expression of LUAD-unfavorable genes, the TCGAbiolinks package (Mounir et al., 2019) was used to download and scan the alteration statuses of LUAD-unfavorable genes. In addition, we randomly selected 10 genes from LUAD-unfavorable gene set and scanned the Human Protein Atlas^4^ (Colwill et al., 2011) web tool to validate whether the LUAD-unfavorable genes are upregulated at protein level, compared with normal lung tissue.

## Results

### Multiple Genes Were Defined as LUAD-Development Characteristic Genes

**Figure 2:**
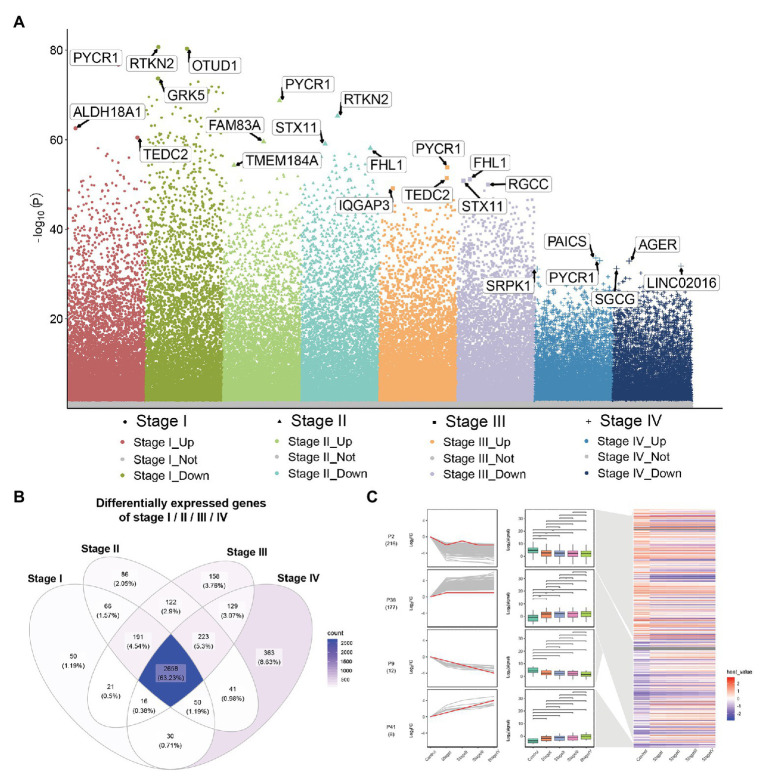
Differential expression gene analysis and short time-series expression miner (STEM) analysis. **(A)** Manhattan plot showed differentially expressed genes (DEGs) in different stage of lung adenocarcinoma (LUAD). Genes with significant differences are highlighted. **(B)** Common DEGs in LUAD stage I–IV. **(C)** STEM results. Line plots (left panels) and box plots (right panels) are used to show fold changes (log2 scale) and absolute expression levels (log2 scale), respectively. In each line plot, one representative gene is highlighted in red.

Compared to normal lung tissue samples, there were 3,082 DEGs in stage I LUADs, 3,437 DEGs in stage II LUADs, 3,518 DEGs in stage III LUADs, and 3,510 DEGs stage IV LUADs (Figure 2A). It indicated that the gene expression patterns were various with the development of LUAD. A total of 2,658 common DEGs was in stage I-IV LUADs (Figure 2B). Among of them, 185 DEGs were gradually upregulated and 237 DEGs were gradually downregulated with the development of LUAD, which maybe play a crucial role in the LUAD development. The result of STEM demonstrated that two gene clusters were significantly upregulated, while two gene clusters were significantly downregulated with the development of LUAD (Figure 2C).

### LUAD-Development Characteristic Genes Were Associated With LUAD Prognosis

**Figure 3:**
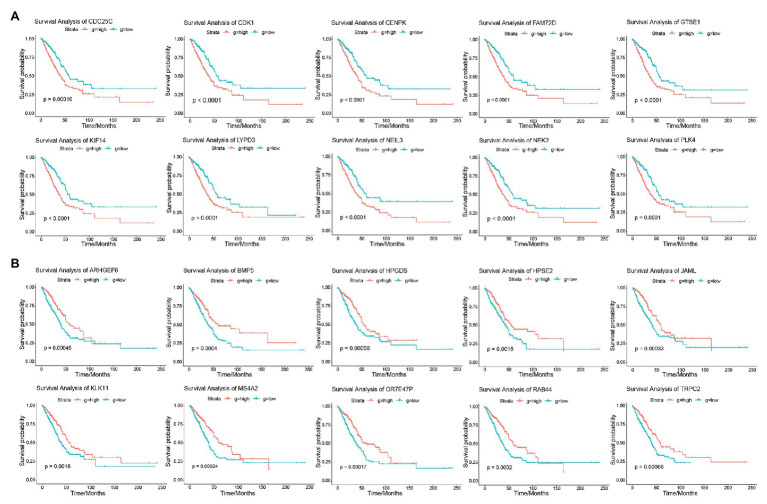
Survival analysis. **(A)** Survival curves of 10 genes most significantly correlated with LUAD in LUAD-unfavorable gene set. **(B)** Survival curves of 10 genes most significantly correlated with LUAD in LUAD-favorable gene set.

The result of survival analysis showed a total of 84 LUAD-development characteristic genes that are gradually upregulated with the development of LUAD and associated with poor prognosis, while a total of 39 LUAD-development characteristic genes that are gradually downregulated with the development of LUAD and associated with good prognosis (Table 1). This means that not all LUAD-development characteristic genes are associated with the prognosis of LUAD. In the LUAD-unfavorable gene set, NEK2, CENPK, CDC25C, PLK4, LYPD3, FAM72D, NEIL3, GTSE1, CDK1, and KIF14 were the ten genes with most significant association with poor prognosis (Figure 3A). While in the LUAD-favorable gene set, OR7E47P, MS4A2, RAB44, BMP5, ARHGEF6, JAML, TRPC2, HPGDS, HPSE2, and KLK11 were the ten genes with most significant association with good prognosis (Figure 3B).

**Table 1:** LUAD-unfavorable gene set and LUAD-favorable gene set.

Gene set	Gene symbol
LUAD-unfavorable gene set	ARHGAP11A, ASPM, BLM, C5orf34, CA9, CCNA2, CDC25C, CDC6, CDCA2, CDK1, CENPF, CENPK, CHAF1B, CLSPN, DDX11-AS1, DEPDC1, DNMT3B, DTL, E2F7, ECT2, EGLN3, ESCO2, EXO1, FAM111B, FAM57B, FAM72D, FAM83D, FANCI, FBXO43, GAL, GTSE1, HASPIN, HELLS, HMMR, KIF11, KIF14, KIFC1, KNL1, KNTC1, KREMEN2, KRT6A, KRT81, LINC01269, LOC101929128, LYPD3, MAD2L1, MELK, MIR924HG, MKI67, MYO19, NCAPG, NDC80, NEIL3, NEK2, NUF2, NUSAP1, OIP5, ORC1, ORC6, PAICS, PARPBP, PCLAF, PIMREG, PLK1, PLK4, POLQ, PRC1, PTPRN, RAD51, RRM2, SGO1, SLC2A1-AS1, SPAG5, SPOCK1, TEDC2, TESMIN, TGFBR3L
LUAD-favorable gene set	TICRR, TROAP, TTK, TYMS, UBE2T, UCA1, ZWINT, ACKR1, ADAMTS8, ADGRF5, ARHGEF6, ATP13A4, BMP5, CASS4, CCDC69, CLEC3B, COL6A6, CTSG, FAM189A2, FBP1, FCER1A, FLI1, GCSAML, GIMAP4, GIMAP7, HPGDS, HPSE2, INMT, JAML, KLK11, LSAMP, LY86, MAL, MS4A2, OR7E47P, P2RY12, RAB44, RTN1, SCN2B, SIGLEC17P, SLCO4C1, SPN, TM6SF1, TRPC2, UNC45B, ZEB2

### LUAD-Unfavorable GSVA Score and LUAD-Favorable GSVA Score Are Biomarker of LUAD and LUAD Prognosis

**Figure 4:**
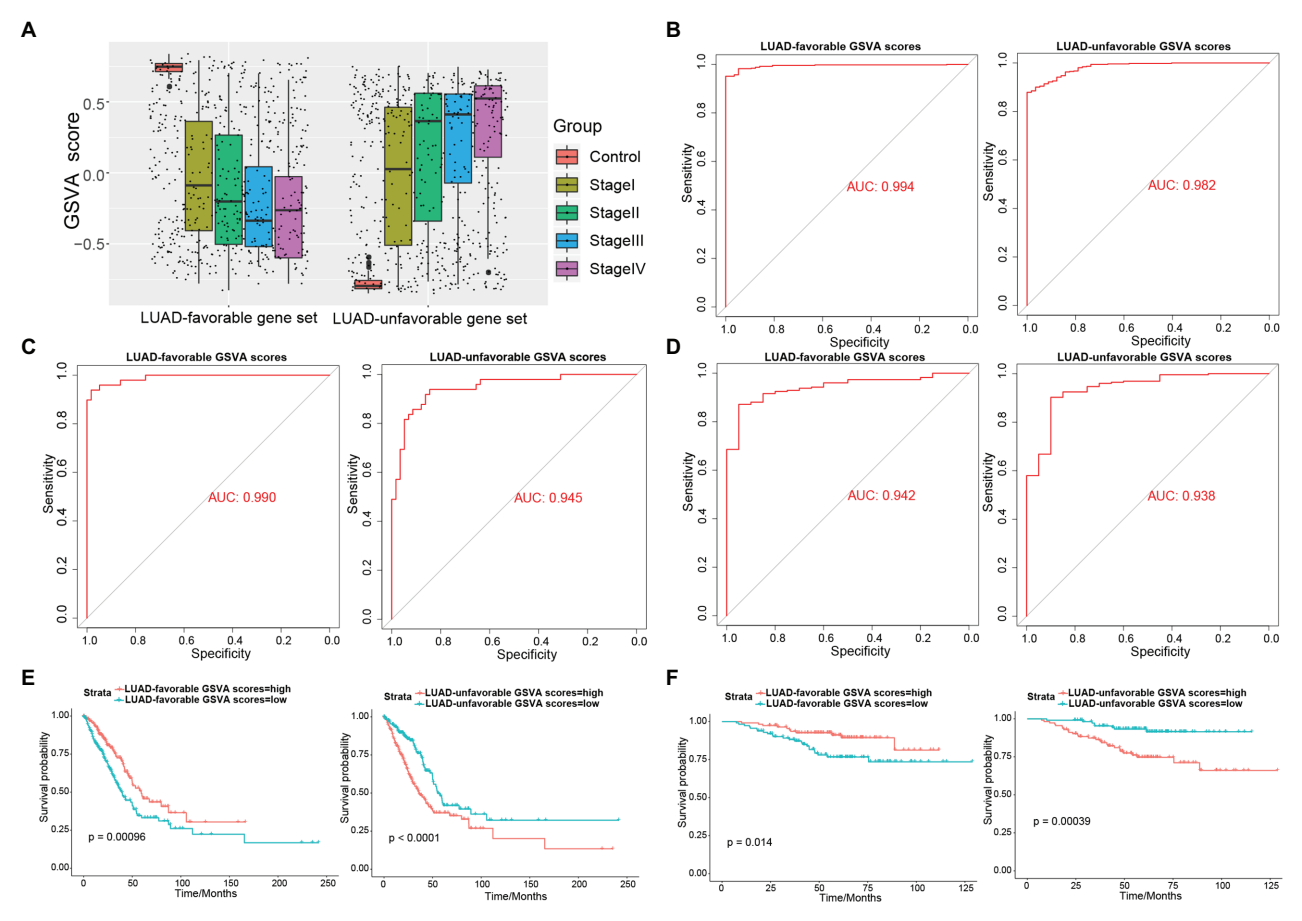
Exploring the diagnostic and prognostic abilities of LUAD-unfavorable gene set and LUAD-favorable gene set. **(A)** The box plots of LUAD-unfavorable gene set gene set variation analysis (GSVA) score and LUAD-favorable gene set GSVA score. **(B)** Receiver operating characteristic (ROC) curves analysis of LUAD-unfavorable gene set GSVA score and LUAD-favorable gene set GSVA score. **(C)** ROC curves analysis of LUAD-unfavorable GSVA score and LUAD-favorable GSVA score in GSE10072. **(D)** ROC curves analysis of LUAD-unfavorable GSVA score and LUAD-favorable GSVA score in GSE31210. **(E)** Survival analysis of LUAD-unfavorable gene set GSVA score and LUAD-favorable gene set GSVA score in LUAD from The Cancer Genome Atlas (TCGA). **(F)** Survival analysis of LUAD-unfavorable GSVA score and LUAD-favorable GSVA score in GSE31210.

As shown in Figure 4A, LUAD-favorable GSVA score was gradually downregulated with the development of LUAD, while LUAD-unfavorable GSVA score was gradually upregulated with the development of LUAD. Moreover, the result of ROC curve analysis indicated that both LUAD-unfavorable GSVA score and LUAD-favorable GSVA score are a biomarker of LUAD with AUC = 0.982 and AUC = 0.994, respectively (Figure 4B). Furthermore, the two GSVA score systems were also validated in GSE10072 (Figure 4C) and GSE31210 (Figure 4D), respectively. According to median GSVA score, all LUAD patients in TCGA were separated into low-score group and high-score group. And both the two GSVA score systems were significantly associated with LUAD prognosis (Figure 4E). Patients with high LUAD-unfavorable GSVA score had worse prognosis, while patients with high LUAD-favorable GSVA score had better prognosis. Univariate and multivariate Cox analysis showed that the two GSVA score systems were the independent factors for LUAD prognosis compared with clinicopathological features (Tables 2 and 3). Moreover, the two GSVA score systems were also significantly associated with LUAD prognosis in GSE31210 (Figure 4F).

**Table 2:** Univariate and multivariate analyses of LUAD-unfavorable GSVA score.

Factor	Univariate Cox analysis			Multivariate Cox analysis		
	*β*	*p*	HR (95% CI)	*β*	*p*	HR (95% CI)
Gender (female/male)	0.025	0.867	0.763–1.378			
Age (>65 years/≦65 years)	0.178	0.243	0.886–1.610			
T stage (T3–4/T1–2)	0.821	0.000	1.543–3.346	0.589	0.016	1.114–2.914
Lymph node stage (N2–3/N0–1)	0.818	0.000	1.582–3.243	0.121	0.757	0.523–2.437
Metastasis (M1/M0)	0.749	0.006	1.234–3.626	0.109	0.799	0.482–2.580
Pathological stage (III–IV/I–II)	0.967	0.000	1.924–3.592	0.509	0.211	0.749–3.695
LUAD-unfavorable GSVA score (high/low)	0.614	0.000	1.365–2.500	0.575	0.002	1.230–2.565

**Table 3:** Univariate and multivariate analyses of LUAD-favorable GSVA score.

Factor	Univariate Cox analysis			Multivariate Cox analysis		
	*β*	*p*	HR (95% CI)	*β*	*p*	HR (95% CI)
Gender (female/male)	0.025	0.867	0.763–1.378			
Age (>65 years/≦65 years)	0.178	0.243	0.886–1.610			
T stage (T3–4/T1–2)	0.821	0.000	1.543–3.346	0.557	0.028	1.062–2.869
Lymph node stage (N2–3/N0–1)	0.818	0.000	1.582–3.243	0.420	0.254	0.739–3.134
Metastasis (M1/M0)	0.749	0.006	1.234–3.626	0.263	0.518	0.586–2.886
Pathological stage (III–IV/I–II)	0.967	0.000	1.924–3.592	0.365	0.361	0.659–3.152
LUAD-favorable GSVA score (high/low)	−0.489	0.001	0.454–0.828	−0.434	0.017	0.453–0.926

### The Differential Expression of LUAD-Unfavorable Gene May Not Result From Mutation

**Figure 5:**
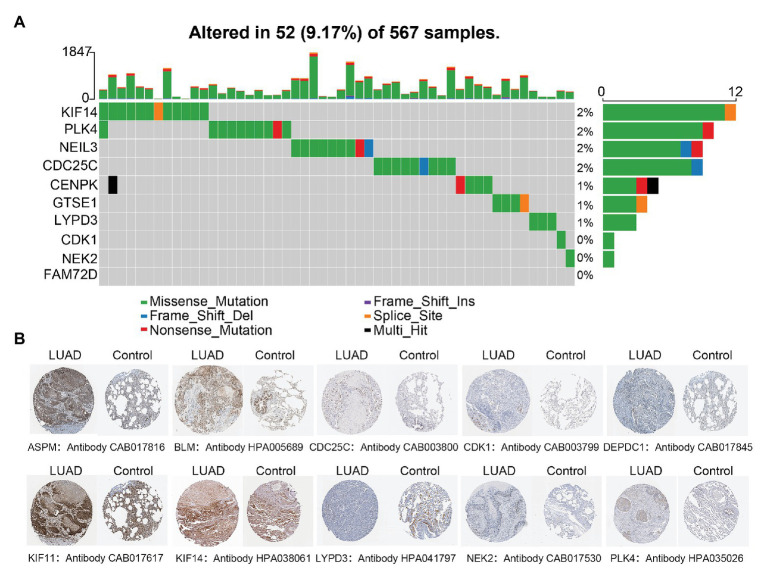
Genetical alteration analysis and immunohistochemistry analysis. **(A)** Genetical alteration analysis of LUAD-unfavorable genes. **(B)** Identification of LUAD-unfavorable gene at protein level. Lung cancer samples are on the left and normal lung tissue samples are on the right.

Only 52 (9.17%) of 567 samples had an alteration in one or several LUAD-unfavorable genes and most samples did not have genetical alteration (Figure 5A). Moreover, compared with normal lung tissue, ten genes (ASPM, BLM, CDC25C, CDK1, DEPDC1, KIF11, KIF14, LYPD3, NEK2, and PLK4) of LUAD-unfavorable gene set were included in The Human Protein Atlas and were highly expressed in LUAD (Figure 5B).

### LUAD-Unfavorable Genes Involved in Multiple Cancer-Related Pathways

**Figure 6:**
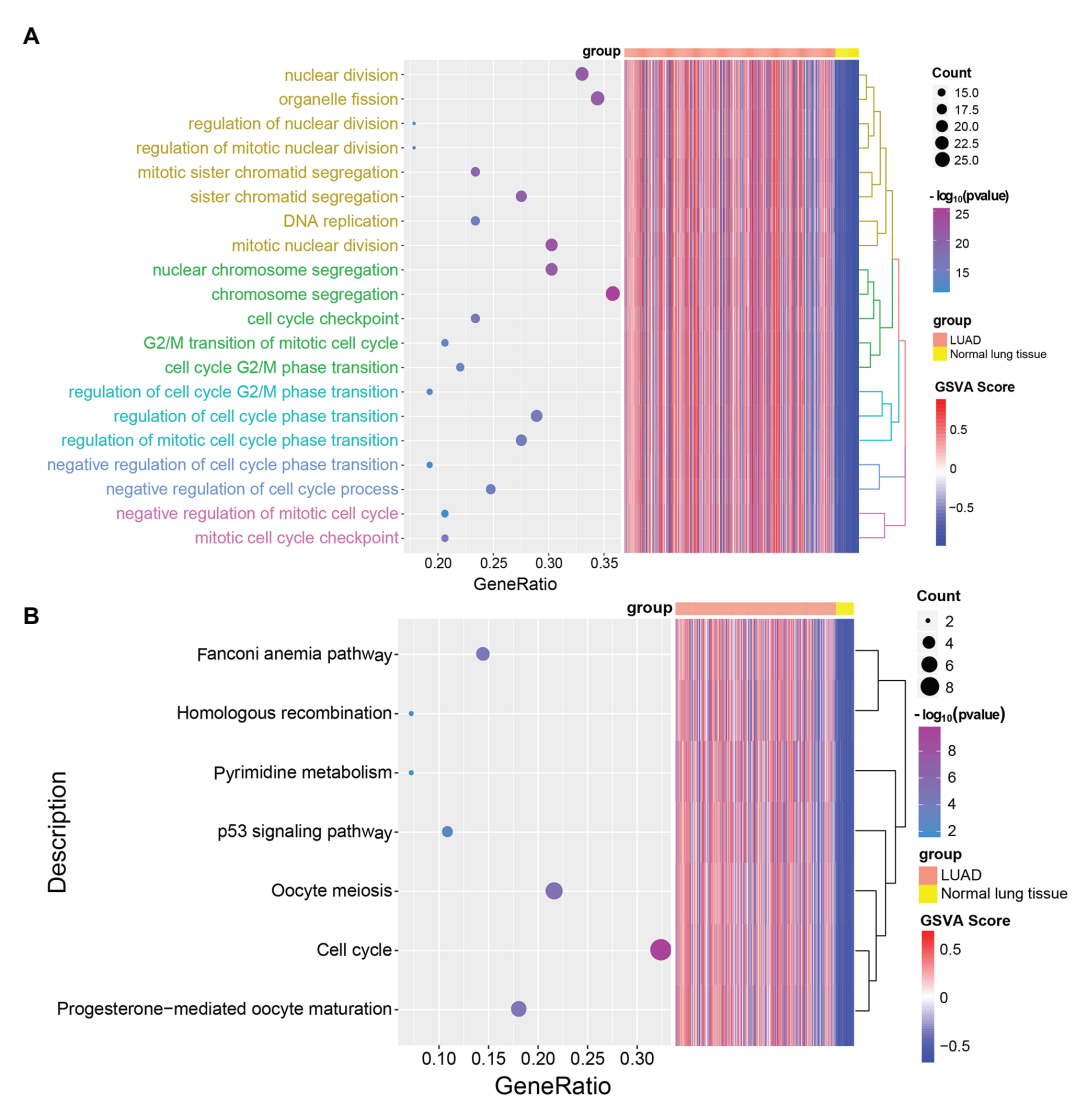
Gene Ontology (GO) biological process (BP) and Kyoto Encyclopedia of Genes and Genomes (KEGG) pathways enrichment analysis of LUAD-unfavorable genes. **(A)** Biological process of LUAD-unfavorable genes. **(B)** KEGG pathway analysis of LUAD-unfavorable genes.

In order to explore the biological functions of LUAD-unfavorable genes, LUAD-unfavorable genes were performed functional enrichment analysis. The results showed that these genes are mainly related to nuclear division, organelle fission, mitotic nuclear division, nuclear chromosome segregation, and chromosome segregation (Figure 6A). Moreover, LUAD-unfavorable genes were significantly involved in many pathways, such as Fanconi anemia pathway, p53 signaling pathway, Oocyte meiosis, Cell cycle, and Progesterone-mediated oocyte maturation (Figure 6B).

## Discussion

In the world, lung cancer is the main cause of cancer-related death. Even with surgical treatment, the recurrence rate of lung cancer still is very high (Scott et al., 2007). Therefore, it is of great significance to explore biomarkers which can accurately diagnose lung cancer and predict prognosis for the treatment and management of lung cancer. A large number of studies have shown that abnormal expression of genes in lung cancer (including LUAD) is closely related to prognosis, and can be used as a potential biomarker of prognosis (Xu et al., 2013; Cui et al., 2015; Giatromanolaki et al., 2015).

In the present study, we found a number of genes were differentially expressed in LUAD different stages. This indicated gene expression patterns were various with the LUAD development. Compared to normal lung tissue, a gene may be differentially expressed in early LUAD but not in advanced stage. We identified 422 LUAD-development characteristic genes, including 185 genes gradually upregulated and 237 genes gradually downregulated with LUAD-development. The development of LUAD results from synergistic effects of multiple genes. Notably, not all LUAD-development characteristic genes are associated with the prognosis of LUAD. LUAD-unfavorable gene set contained 84 gradually upregulated DEGs and LUAD-favorable gene set contained 39 gradually downregulated DEGs. Unsurprisingly, previous studies have suggested that some of them are associated with LUAD development. NEK2 is overexpressed in a variety of malignant tumors and is closely related to tumor drug resistance, rapid recurrence, and poor prognosis (Zhou et al., 2013; Fang and Zhang, 2016; Li et al., 2017). KIF14 has also been found to be associated with poor prognosis in a variety of cancers (O’Hare et al., 2016; Zhang et al., 2017). While in the LUAD-favorable gene set, genes which were significantly associated with LUAD survival included OR7E47P, MS4A2, RAB44, BMP5, ARHGEF6, and KLK11. Among them, KLK11 was found to be a diagnostic and prognostic indicator of NSCLC (Xu et al., 2014). These result confirmed the possibility that the LUAD-unfavorable gene set and LUAD-unfavorable gene set can be used as a prognostic model for LUAD.

All samples were calculated LUAD-unfavorable GSVA scores and LUAD-favorable GSVA scores. This is obviously different from the gene signatures in other previous studies (Li et al., 2014; Shi et al., 2018; Liu et al., 2019). In the previous studies, a gene often got a coefficient from a Cox regression analysis or other method in the training set. However, due to the limitations of the sample size and the heterogeneity of the tumor, we may never know the true coefficient of a gene. Therefore, GSVA was used to score individual samples against gene sets (LUAD-unfavorable gene set and LUAD-favorable gene set) in our study. ROC curve analysis suggested that both LUAD-unfavorable GSVA score and LUAD-favorable GSVA score exhibited strong diagnostic capacity of LUAD and which was verified in other two independent data sets. Univariate and multivariate Cox regression analysis suggested that LUAD-unfavorable GSVA score and LUAD-unfavorable gene set were independent prognostic factors for LUAD’s overall survival. This result was also verified in an independent data set.

Moreover, we found that the mutation rate of most genes is very low, indicating that the differential expression of genes may not be caused by mutation. Additionally, functional enrichment analysis indicates that LUAD-unfavorable genes are significantly involved in p53 signaling pathway, Cell cycle, and other pathways. It is suggested that LUAD-unfavorable genes may be involved in the occurrence and development of LUAD through these pathways. However, further studies are needed to investigate and validate the functions of these genes.

In the present study, although we provided new insights into the LUAD prognostic stratification system, several limitations were notable. Firstly, the two gene sets may be too large. Their application to the clinic still needs to wait for further decline in sequencing costs. Secondly, the synergy between the genes of these two gene sets to promote LUAD development still requires molecular experimental validation.

## Conclusion

In conclusion, we identified and validated two LUAD-development characteristic gene sets that not only have diagnostic value but also prognostic value. It may provide new insight for further research on LUAD.

## Data Availability Statement

All datasets presented in this study are included in the article/supplementary material.

## Author Contributions

CL and XL conducted the experiments. HS and DL designed the experiments and wrote the paper. All authors contributed to the article and approved the submitted version.

### Conflict of Interest

The authors declare that the research was conducted in the absence of any commercial or financial relationships that could be construed as a potential conflict of interest.

## Footnotes

^1^https://www.cancer.gov/

^2^https://www.ncbi.nlm.nih.gov/geo/

^3^https://CRAN.R-project.org/package=survival

^4^https://v15.proteinatlas.org/
